# Complex fate of paralogs

**DOI:** 10.1186/1471-2148-8-337

**Published:** 2008-12-18

**Authors:** Radek Szklarczyk, Martijn A Huynen, Berend Snel

**Affiliations:** 1 Centre for Molecular and Biomolecular Informatics, NCMLS, Radboud University Medical Centre, PO Box 9101, 6500 HB Nijmegen, the Netherlands; 2 Bioinformatics, Department of Biology, Faculty of Science, Utrecht University, Padualaan 8, 3584 CH, the Netherlands; 3 Academic Biomedical Centre, Utrecht University, Yalelaan 1, 3584 CL Utrecht, the Netherlands

## Abstract

**Background:**

Thanks to recent high coverage mass-spectrometry studies and reconstructed protein complexes, we are now in an unprecedented position to study the evolution of biological systems. Gene duplications, known to be a major source of innovation in evolution, can now be readily examined in the context of protein complexes.

**Results:**

We observe that paralogs operating in the same complex fulfill different roles: mRNA dosage increase for more than a hundred cytosolic ribosomal proteins, mutually exclusive participation of at least 54 paralogs resulting in alternative forms of complexes, and 24 proteins contributing to *bona fide *structural growth. Inspection of paralogous proteins participating in two independent complexes shows that an ancient, pre-duplication protein functioned in both multi-protein assemblies and a gene duplication event allowed the respective copies to specialize and split their roles.

**Conclusion:**

Variants with conditionally assembled, paralogous subunits likely have played a role in yeast's adaptation to anaerobic conditions. In a number of cases the gene duplication has given rise to one duplicate that is no longer part of a protein complex and shows an accelerated rate of evolution. Such genes could provide the raw material for the evolution of new functions.

## Background

Gene duplication can be a major source of innovation in evolution [1], providing redundancy and additional genetic material to build upon and differentiate. In general, eukaryotic genomes contain a large fraction of gene duplicates, with paralogs stemming not only from single gene or segmental duplications, but, in the case of *S. cerevisiae*, also from a Whole-Genome Duplication event that occurred approximately 100 mln years ago (WGD; [2,3]). Genomic instability and massive gene loss promptly followed WGD and purged most of the newly formed gene copies from the yeast genome, retaining approximately 10% of them [3]. Today, using multiple genomes of related fungal species with conserved synteny, we can unambiguously identify hundreds of gene pairs as WGD paralogs [4] in addition to normal small scale paralogs.

The identification of paralogs of WGD origin, in conjunction with the wealth of data on physical protein interactions and derived maps of protein complexes, puts us in an unprecedented position to test the fate of nascent duplicated genes and to potentially identify cases of duplication of whole complexes. Recently, it has been shown that, after gene duplication, protein interactions can be conserved [5,6]. The data suggested that there exists a stepwise pathway of evolution for such functional modules [6], with duplications of homomeric interactions known to have a significant influence on the evolution of genes [5]. Moreover, it is known that gene duplicates can be found less often among the core components of protein complexes compared to sparse regions of protein interaction network [7]. For our study of the impact of gene duplication on protein complexes, we separated paralogs into two distinct, non-overlapping classes: genes that were duplicated at the WGD event, and non-WGD duplicates detectable by sequence similarity. Dubbed small scale duplications (SSD), these paralogs are the result of the most recent gene duplications, identified per event by employing a best bi-directional hit criterion on all yeast gene pairs (see Methods). From the analysis of the phylogenetic distribution and number of paralogs in related species, it appears that the time of duplication of SSD genes greatly predates the WGD event (see Methods). Both duplication types, WGD and SSD, cover together ~40% of yeast genes, providing a comprehensive overview of these evolutionary events. These two paralog types are already known to differ with respect to their expression pattern [8,9] and synthetic lethality rate [10], by displaying different phenotypic effects when deleted [11] and occurrence across functional classes (e.g., stress responsive genes, [8]). Musso and colleagues [9] show that nearly half of WGD paralogs co-cluster in the same protein complex. Amoutzias and colleagues [12] indicate that whole genome duplication did not change the dimerization specificities of interacting homologs. Here, we show a much more detailed spectrum of evolutionary and functional fates of higher order protein complex subunits. This integrated overview, enables us to quantify the fates with respect to the duplication type and address questions related to protein specialization (subfunctionalization), as well as the emergence of novel functions related to complexes (neofunctionalization).

Our hypotheses were tested on various types of manually curated data: both complexes from MIPS consortium [13], and those annotated by SGD [14]. To avoid a possible bias introduced by manual curation, we also use computationally derived maps of complexes [15,16], reconstruction of which was possible owing to recent mass-spectrometry studies [17,18]. Integration of these datasets allowed us to systematically study the fates of all gene duplicates which are involved in protein complexes.

## Results

### The fates of duplicate genes in complexes

**Figure 1:**
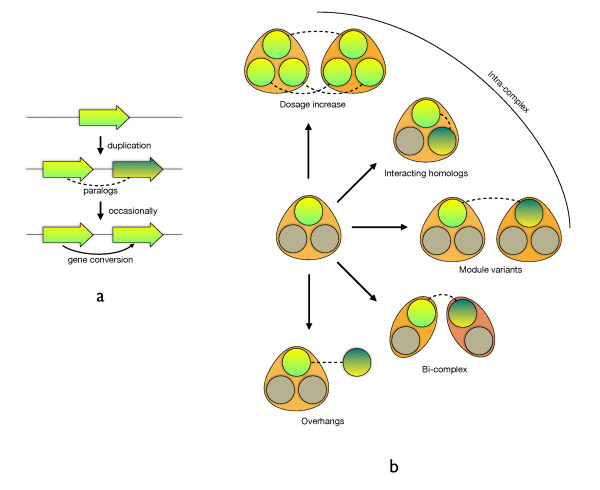
**Complex fate of paralogs**. a) Gene duplication and subsequent divergence, for cytosolic ribosomal proteins (cRP) followed by homogenizing gene conversion events. b) Impact of duplicated proteins on complexes. Intra-complex duplications include dosage increase, interacting homologs and module variants. Dosage increase requires many components of the complex to duplicate simultaneously (as in the case of cRP and the whole genome duplication). For interacting homologs, the two duplicated proteins become physically subunits of the complex (e.g., homomers turning into heterodimers after the duplication). In module variants only one of the two paralogs is present in the protein complex at a given time. Bi-complex paralogs operate in different protein complexes; two possible evolutionary routes are shown. Overhangs do not aggregate with other proteins in a non-transient manner, while their paralogs do.

**Figure 2:**
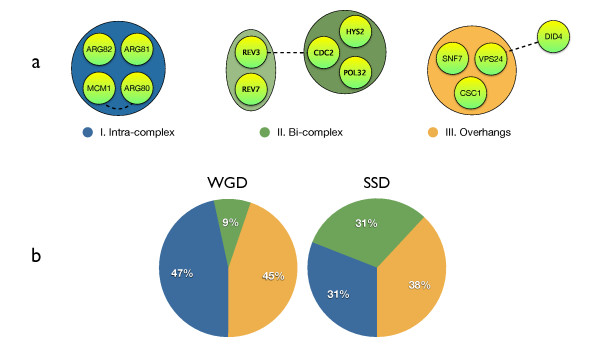
**The roles of paralogs in protein complexes**. a) Shaded areas mark a complex, dashed lines connect paralogs. I) Intra-complex paralogs: when both proteins participate in the same complex; ARG transcription complex includes an intra-complex duplication of genes encoding FUN80 and ARGR1 subunits. II) Bi-complex paralogs: two proteins are involved in different protein complexes; two small complexes are shown: zeta DNA polymerase complex (left) and delta DNA polymerase complex (right). Pair REV3/CDC2 are bi-complex paralogs. III) Overhangs: only one of the paralogs constitutes a subunit of a complex, while its homolog does not aggregate with other proteins in a non-transient manner; Vps4p ATPase transport complex. Here, CHM2 protein (a paralog of DID3) represents an overhang. b) Type of duplication and their contribution to protein complexes: left, whole genome duplication (cytoplasmic ribosomal proteins excluded), and right, small scale duplications. On the pie chart, fractions of all paralog pairs are denoted. Protein complex annotations after SGD consortium.

We carried out a systematic analysis of the fate of paralogs in protein complexes. From our first observations it became clear that the cytosolic ribosomal complex dominates the whole spectrum of gene duplications. In order to prevent this single protein complex to dominate our results, we analyze it separately (see Methods). The fates of other paralogs found within complexes fall into two other categories (Figure 1 and 2). Intra-complex paralogs (I) that are formed when both resulting genes remain within the same protein complex, whereas bi-complex paralogs (II) function within two separate complexes. The third class, which we define as overhangs (III), consists of subunits of complexes with a paralog possessing no association to a known protein complex whatsoever. SSD and WGD paralogs are equally divided over intra-complex and overhang classes, but differ with respect to the bi-complex class: many more SSD paralogs are present in two complexes compared to WGD paralogs (Figure 2b). We discuss this observation below.

### Intra-complex paralogs: retention is an important fate of paralogs within complexes

We observe a very strong preference for both duplicated proteins to function in the same module. Compared to a null model, where proteins are stochastically reshuffled between complexes, intra-complex paralogs are ~40-fold overrepresented (SGD modules, [14]). This preference is similar, and not statistically different for both duplication types (P = 0.97, chi-square test) and holds for other module definitions, including the computationally derived protein complexes from complex co-purification experiments (see additional file 1, Table S1). Paralog retention within the module is thus an important factor in shaping the map of protein complexes.

We thus recover the previously made observation that WGD and SSD paralogs are known to act within the ancestral protein complex after the duplication [7,8]. Further analysis however revealed a wider spectrum of fates in which two paralogs can be involved in a single protein complex, as illustrated in the analysis of the essential yeast chromatin remodeling complex RSC. Owing to the availability of protein-protein interaction data [17,18] we can distinguish between different modes of participation in a single complex. The first, a more "direct" bait-prey interaction mode, occurs when one protein was designated a bait and the other protein co-purified as a prey; this event is characterized by a high spoke value [17]. The second type of interaction, a prey-prey interaction, can be detected when two prey proteins were co-purified with the same bait in two independent purification experiments and does not provide the evidence for the two proteins to co-occur in the same protein complex at a given time. Hence, we were able to specify the following intra-complex fates:

#### Ia. mRNA dosage effect

Undoubtedly, the single most abundant fate of paralogs is the mRNA dosage increase of cytosolic ribosomal proteins (CRPs), as more than half of 200 intra-complex proteins are CRPs (see Table 1 and Methods for details). Their coordinate expression is primarily accomplished at the transcriptional level [19], signified by high expression correlation between the paralogs (our results show Spearman's rank correlation coefficient of 0.8). It is a markedly different type of evolutionary innovation from other classes, as paralogous CRPs, due to undergoing gene conversion [20], are highly similar in their protein sequence (see Methods).

**Table 1:** Paralogs in complexes.

	Intra-complex		Bi-complex		Overhangs
	**# proteins**	observed/expected*	**# proteins**	**observed/expected**	**# proteins**

**Paralogs**	216	46 (P < 10^-3^)	62	0.3 (P < 10^-3^)	58

**Comments**	110 dosage increase 24 interacting homologs 54 module variants		Mostly old duplications, 5× less WGD paralogs (P < 4*10^-4^)		

We note a strong over-representation and a statistically significant, many-fold enrichment (intra-complex) or depletion (bi-complex) compared to a null model for paralog evolution.

#### Ib. Interacting homologs

This subclass consists of paralogs both present in a protein complex at the same time. Using protein-protein interaction data we identified 24 intra-complex paralogs with a bait-prey interaction type, signified by a high spoke value of least 5 (see Methods for details). This class is exemplified by the RSC3/RSC30 pair from RSC chromatin remodeling complex, known to form a stable heterodimer [21]. This kind of relationship between paralogs is likely to result from an ancestral homodimer, where a paralogous replacement of the dimer's components took place [5]. Strong positive co-expression (Spearman's correlation coefficient of 0.4), even though weaker than the tightly co-regulated CRPs, provides additional clues for simultaneous presence of both proteins in the functional module. Homomers undergoing this evolutionary route are probably the classic view on how two paralogs are involved in the same protein complex, as exemplified by the F1-ATPase alpha and beta subunits [22].

#### Ic. Module variants

This perhaps somewhat less explicitly recognized category embraces paralogs with a seemingly intrinsic contradiction: operating within the same "complex", yet never present together with only a prey-prey evidence for their interaction. Such mutually exclusive presence implies existence of different variants of the same complex. To assign proteins to the module variants class, we select intra-complex paralogs with no evidence of direct interaction. That includes paralogs never purified together, or with a negative spoke value (see Methods for details). Our analysis yields 54 intra-complex paralogs that belong to this class. Lower co-expression of these genes, likely resulting from the functional role undertaken by paralogs, confirms that these subunits are alternatively present in a module, thus not required to operate simultaneously (average co-expression Spearman's correlation 0.2, statistically different from other classes, one sided Wilcoxon ranked sum test, P < 0.02). More divergent expression also suggests a mechanism of control of complex activity by conditional assembly (analogous to just-in-time assembly for cell cycle complexes, [23]).

The literature provides many tantalizing clues to the conditions in which these alternative assemblies (module variants) may operate. For example, the WGD paralogs TPK1/TPK3 have a negatively correlated co-expression (Spearman's correlation coefficient -0.3), although they are both part of the cAMP-dependent protein kinase complex. Glucose-induced hyperaccumulation of cAMP was observed in exponential-phase glucose-grown cells of the TPK1-deficient but not the TPK3-deficient strain [24]. Moreover, investigation of mitochondrial respiration by in vivo 31P nuclear magnetic resonance spectroscopy showed the tpk1- and not tpk3-mutant, to be defective in glucose repression [24]. Another clue hinting at specialization for carbon source utilization comes from the WGD paralogs COX5a/COX5b. The two subunits are encoded by divergent sequences, but are functionally interchangeable forms of yeast cytochrome c oxidase subunit V [25]. COX5a/COX5b paralogs are oppositely regulated (Spearman's expression correlation -0.3) and are known to be expressed in a mutually exclusive manner under aerobic (COX5a) and anaerobic conditions (COX5b) [26]. Taking all module variants together, we observe their enrichment among WGD paralogs (P < 0.02) and also module variants are 2.5 less likely to be essential when considering viability of single-gene knockouts (P < 0.01, Table 2). This apparent redundancy of module variants in rich medium does not exclude their possible contribution to cell's survival in other growth media. To test this hypothesis we analyzed the data on growth rates of yeast deletion mutants in nine fermentable and non-fermentable substrates [27]. 12 module variants and none of interacting homologs show differential and partly complementary pattern of growth rates in various carbon sources (Table 3). Additionally, phenotype of single-gene deletions of module variants does not correlate between paralogs (average correlation coefficient ~0.1) when tested in hundreds chemical and environmental stress conditions [28]. Together, all this evidence leads us to suggest that the WGD event might have facilitated the evolution of anaerobic fermentation in *S. cerevisiae *via introduction of many specialized module variants.

**Table 2:** Type of intra-complex paralogs and viability of single-gene knockouts in rich medium.

Intra-complex duplication type	Fraction essential
**Interacting homologs**	50% (12/24)

**Module variants**	19% (10/54)

Average*	32% (71/225)

*) calculated among all paralogs involved in modules.

**Table 3:** Deletion mutants of module variants exhibit differential growth patterns when cultured on various carbon sources.

Module variant paralogs	Deletion phenotype
COX5A/COX5B	COX5A knockout: reduced fitness when no glucose

KIP1/CIN8	CIN8 knockout: unrestricted growth only on glycine

BUL2/BUL1	BUL1 knockout: reduced fitness on ethanol

DID4/VPS24	DID4 knockout: severely reduced growth on lactate

NOT5/NOT3	NOT3 knockout: severely impaired growth on glycine NOT5 knockout: growth severely impaired in all conditions tested

REG2/REG1	REG1 knockout: limited growth on glucose

### Bi-complex paralogs: proteins functioning in different complexes

As opposed to intra-complex paralogs, where both proteins function in the same module, bi-complex paralogs each participate in distinct ones. Depending on the map of protein complexes, 44 or more genes fall into this category (see Table 1 and additional file 1, Table S2). We confirmed the lack of interaction between this type of paralogs with protein-complex purification data (only two out of 31 pairs were ever purified together, significantly less than intra-complex paralogs, Fisher exact test P < 4e-5, odds ratio 15).

Interestingly, for bi-complex paralogs, a significant difference between WGD and SSD duplicates can be seen. The majority of them are SSD duplicates (see additional file 1, Table S2). This strong bias, with SSD constituting more than 80% of the bi-complex class, contrasts with handful of WGD paralogs split between different complexes. We propose this to be an effect associated with the age of duplication. The lion's share of SSD paralogs not only predate the WGD event but are older than the divergence with *S. pombe*. While none of the eight post-*S. pombe *SSD duplications is bi-complex, three duplications are intra-complex (see additional file 1, Table S5), a hint that not the type of duplication (SSD versus WGD), but its age has a greater influence on the paralog's fate. Over extended evolutionary time since the ancient duplication of majority of SSD paralogs, many specialized (subfunctionalization), join or even established a new complex (neofunctionalization), ultimately leading to the bi-complex relationship. The conservative nature of interaction evolution after gene duplication is confirmed by the underrepresentation of bi-complex paralogs, compared to a null model where proteins are free to change their complex following the duplication (Table 1 and additional file 1, Table S2).

**Figure 3:**
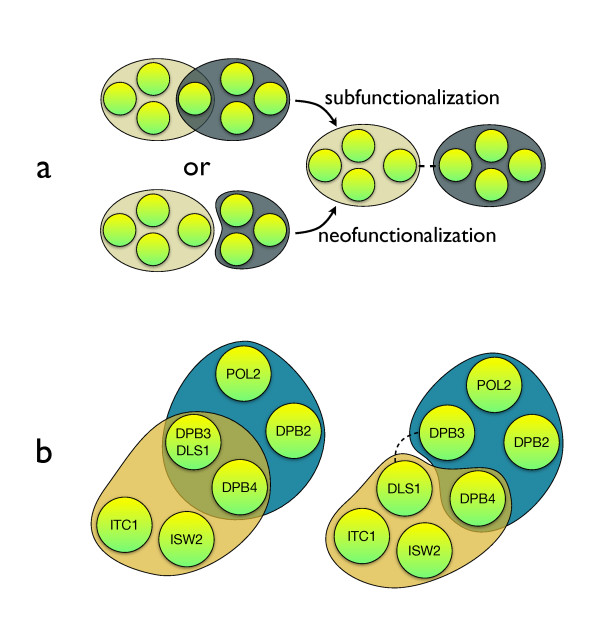
**Evolution of bi-complex paralogs**. a) Bi-complex paralogs (right) are potentially cases of sub-functionalization (upper route; bi-complex protein becomes bi-complex paralogous pair) neo-functionalization (single protein in which one paralogous copy is added to another complex). b) A possible evolutionary scenario for a gene duplication event involving epsilon DNA Polymerase and CHRAC complexes. Left, proposed pre-duplication relationships between complexes (ancestral DPB3/DLS1 gene is pictured here before its duplication). Right, the map of modules after a DLS1-DPB3 protein duplication (current *S. cerevisiae *protein complexes) suggests subfunctionalization.

What evolutionary route would lead to the emergence of bi-complex paralogs? Two possible scenarios are shown in Figure 3a. Unfortunately, we do not have a map of protein complexes prior to gene duplications and we have to rely on the indirect evidence of ancestral state. Manual inspection of the five cases of WGD bi-complex paralogs indicates that their complexes overlap (e.g. still posses shared subunits) and the bi-complex paralogs tend to show some genetic redundancy. For example, the DPB4 subunit shared between epsilon DNA Polymerase and CHRAC complexes (see Figure 3b), is indication that before the WGD event the ancestral DPB3/DLS1 subunit functioned, similarly, in the two complexes (see additional file 1, "Tracing evolutionary past of paralogs"). Based on these clues we conclude that the most common evolutionary scenario for bi-complex paralogs is as follows: a single ancestral protein operated in two independent complexes and a duplication event allowed the respective copies to specialize and split their roles. This preferred route would be an indication of subfunctionalization, rather than of neofunctionalization.

### Examples of whole-complex duplications

**Figure 4:**
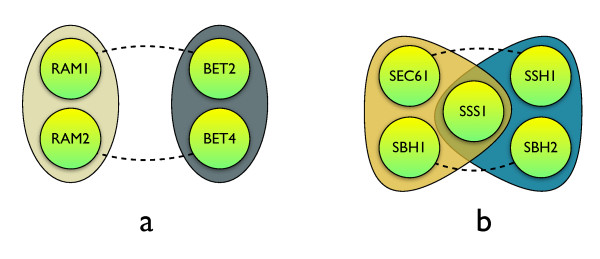
**Examples of a whole-complex duplication**. a) Farnesyltransferase complex (left) prenylates a-factor mating pheromone and Ras proteins, is required for their membrane localization [48]. Geranylgeranyltransferase complex (right) catalyzes the prenylation of Rab proteins, is required for membrane attachment and their subsequent biological activity [49], and is indispensable for vesicular transport between the endoplasmic reticulum and the Golgi. b) Endomplasmic reticulum membrane translocons Sec61 (left) and Ssh1 (right).

A dramatic result of numerous bi-complex duplications could be a duplication of a whole complex. Particularly, a whole-genome duplication, by simultaneous duplication of all subunits, provides the necessary material to make a "carbon copy" of complexes. Instead, we observed a statistical under-representation of bi-complex paralogs. Nevertheless, single events of whole-complex duplication can be identified. For example, the farnesyltransferase and geranylgeranyltransferase complexes both consists of two subunits: RAM1-RAM2 and BET2-BET4 respectively, originating as ancient, pre-WGD duplications of RAM1/BET2 and RAM2/BET4 ancestral genes. Although it is not know which was the original complex (or whether the ancestral complex served both functions) this suggests that two stepwise small-scale duplications occurred, ultimately copying the whole complex that went on to evolve distinct functions (see Figure 4a).

Another case of whole-complex duplication involves a three-protein Sec61 complex (also referred to as a translocon, Figure 4b). This essential complex forms a channel in the ER membrane and mediates translocation of secretory and membrane proteins into the ER and also retrograde transport of misfolded proteins to the cytoplasm for degradation [29,30]. The complex has duplicated in the course of evolution to form an Ssh1 translocon complex [31]. The Ssh1 complex, a result of small scale duplications, also functions in co-translational import to the endoplasmic reticulum (an essential paralogous subunit Sec61p plays a post-translational role as well), and is required for normal growth rates.

### Overhangs – lone paralogs

The final class of paralogs are overhangs, proteins without an association to a functional module, but with a paralog known to be involved in a protein complex (Figure 1). For SGD protein complexes, we found 58 such proteins, with no significant difference in contributions of WGD and SSD duplication types for most of the protein complex maps (see additional file 1, Table S3). Validation with TAP protein complex purification data shows virtually no association of overhangs with their paralog's module (average interaction spoke value for overhangs is 0.06 compared to 2.6 for their "in-module" partners). Additionally, compared to their paralogs, less functional data about overhangs is available. Perhaps predictably, 11/58 overhangs genes are unnamed genes (i.e. not described in a scientific publication), compared to all of their paralogs being named (Fisher's exact test, P < 0.01). Naming roughly reflects the state of our knowledge about the gene, and we further observe absence of annotation in Molecular Function (P < 0.02) and Biological Process (P < 0.01) classes of GO.

To further validate the role overhangs play in the cellular processes we counted the essential genes (inviable null mutants) among them. Even after excluding unnamed genes from this analysis, we have only 4/48 essential overhangs, compared to 17/58 of their in-module paralogs (Fisher's exact test P < 0.01, odds ratio 4.5). This corroborates with the hypothesis of Hart et al. [15] that essentiality is a product of the protein complex, rather than the individual protein. We conclude that overhangs play a much less important role in cell biology, at least in the rich medium conditions in which most of the functional studies are performed.

We observed that overhangs are less constrained by evolution on the sequence level. For WGD overhangs, we compared amino acid identity levels of paralogs against their *Kluyveromyces waltii *ortholog (there is a single ortholog in *K. waltii*, as this species diverged before the WGD event). The amino acid sequence of overhangs diverges significantly faster compared to their in-module paralogs (34% vs 40% global amino acid identity, one sided paired Wilcoxon signed rank test P < 0.02). We therefore conclude that being a part of the protein complex imposes certain constraints on divergence, and the process of orphaning coincides with an increased rate of sequence evolution.

A higher rate of protein sequence evolution and almost complete loss of interactions with an ancestral protein complex are manifestations of rapid functional divergence. The orphaned proteins are involved in different cellular processes: e.g., an overhang SSD1 (suppressor of SIT4 deletion, YDR293C), interacts with a TOR pathway, and functions in sustaining cell wall integrity [32], while its paralog DIS3 is a catalytic component of exosome [33], also involved in mitotic control [34]. We measured the degree of function divergence of overhangs and their paralogs. Using semantic similarity based on Gene Ontologies (see Methods), genes were assigned values between 0 (for different function) and 1 (highly similar or identical function). We observe a rapid divergence of functionality for overhangs (additional file 1, Figure S1). This analysis hints to the overhangs as one of nature's methods to gene neofunctionalization.

## Discussion and conclusion

For the paralogs participating in different complexes (bi-complex paralogs), we see a quantitative difference between duplicates of different age, with only a minority of bi-complex paralogs stemming from WGD. We attribute the higher representation of SSD paralogs to the time of the duplication. The mixture of functional data and the knowledge of their evolutionary history enabled us to reconstruct the evolutionary past of WGD paralogs. As bi-complex paralogs might have potentially undergone either neo- or subfunctionalization (see Figure 3a), we suggest, based on the examination of the association between complexes, that bi-complex paralogs could be examples of function specialization in the protein interaction network.

As observed in [8] there is no overrepresentation of whole modules being duplicated at the WGD event. A massive duplication is a unique opportunity for an organism to replicate components of its cellular machinery (e.g., protein complexes) and let it subsequently evolve independently, with each complex following its own evolutionary path. And even though it appears that gene pairs [35] and transcriptional network show features of partitioning into heavily intra-connected, but sparsely inter-connected clusters [36] at the protein complex level we did not observe large-scale duplications. Is it maybe that the ancestor of *S. cerevisiae *around 100 mln years ago had a chance to duplicate complexes as a whole, but missed the unique opportunity? Certainly the case of cytoplasmic ribosomes is an example of the ancestral yeast cell taking advantage of WGD event and doubling the subunit count in this protein complex. In fact, the completeness of the duplication of the cytoplasmic ribosome (both the small and large subunit) allowed the cell to maintain required equimolar concentrations of CRPs [37,38] while doubling the gene repertoire, a goal not attainable by stepwise module growth and multiple small duplications.

**Figure 5:**
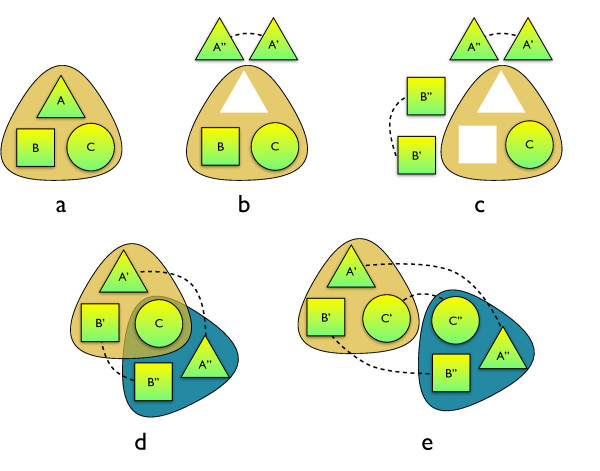
**A possible evolutionary scenario for incremental duplication of whole complexes**. a) Ancestral three-subunit protein complex before duplications. b) Intra-complex duplication, gives rise to paralogous proteins, mutually exclusive as a part of the complex. Two module variants arise, with either A' or A" as one of subunits. c) Another intra-complex duplication makes possible assembly of up to four distinct module variants. d) Paralogs co-evolve to work together, specializing to accept only one of the interacting paralogs. This gives rise to more accentuated module variants, which now can be interpreted as separate complexes with a shared subunit. e) Eventually, all subunits may get duplicated, creating two independent protein complexes.

Many parallel functional modules has been identified across all life kingdoms, including helicases and heat shock proteins in yeast [39]. Nevertheless, the absence of complete complex duplication at the WGD event (with a notable exception of CRPs) indicates that a stepwise duplication of modules [6], rather than whole-complex duplications, is a major mode of protein complex evolution in eukaryotes. Subunit-by-subunit module expansions amend the cellular machinery with the introduction of module variants. Subsequent duplications may give rise to bi-complex paralogs, which can be seen as intermediate phases on the evolutionary path leading to whole-complex duplication (see Figure 5). The process may be accompanied by attachment of additional subunits to one of the complexes or a differential loss of existing ones.

In eukaryotes a single protein events (loss, duplication or gain) dominate the evolution of functional modules. Even though here we do not quantify the prokaryote/eukaryote difference, scientific literature indicates that multiple copies of a protein complex can be found in bacteria. In the case of Complex I submodules, homologs of some of the recruited proteins already performed a function together previous to their involvement in the new pathway, and were duplicated in parallel of shortly after each other. This type of modular evolution in prokaryotes includes a duplication of, sometimes sizable, complexes: we know that a formate hydrogenlyase complex (FHL) of *E. coli *is in close evolutionary relation to Complex I [40]. Additionally, a duplication-prone FHL complex can be found in two copies in *E. coli *(FHL-1 and FHL-2), differing by only three subunits [41]. This observations lead to the hypothesis that appearance of copies of protein complexes in prokaryotes may be associated with the operon structure. The whole module encoded by an operon could duplicate by means of a single, segmental duplication. Alternatively, related complexes could evolve independently in different bacterial species and then be brought together by the horizontal gene transfer of the whole operon. Either way separate, independently functioning copy of a module could, for example, become recruited as a submodule of a bigger protein complex [42]. Interestingly, both *E. coli *FHL complexes are encoded by two operons, Hyf and Hyc [41].

**Figure 6:**
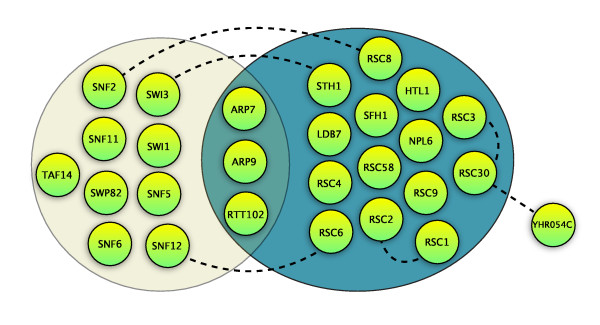
**The RSC protein complex (right) together with the related SWI/SNF complex (left) exhibit intricate homologous relationships between complexes' components**. SWI/SNF and RSC complexes share 3 common proteins (ARP7, ARP9, RTT102). Additional three genes from each complex are paralogs (dashed lines), suggesting series of duplications (this includes a WGD pair RSC6-SNF12). For these bi-complex paralogs, it is very likely that the pre-duplication/ancestral forms of these genes were participating in both complexes, and the duplication event allowed paralogs to assume more specific roles in either complex. Zinc-cluster domain containing RSC3 and RSC30 are paralogs found in RSC complexes in equal proportions [43] and are known to interact physically, forming a stable heteromeric complex [21]. Despite operating as a heterodimer (possibly within the RSC complex as well), the two genes have different functions, with only RSC3 being an essential gene. The intra-complex paralog pair originated at the WGD event. At a given time, both proteins can be found in the complex, hence increase the subunit count by one above the initial complex, given that the ancestral interaction was probably a homomeric one. RSC1 and RSC2 (WGD paralogs), associate with the complex in a mutually exclusive manner [47] (confirmed by a low spoke value of 2.1 compared to average 5.0 for other subunits). Strains deficient in either RSC1 or RSC2 do not display significant growth deficiency, while synthetic lethality of paralogs suggests a certain degree of function redundancy [47]. A similar, but non-identical function of the two paralogs is revealed by differences in phenotypes of yeast strains with RSC1 (growth deficiency in hydroxyurea) or RSC2 deletions (temperature sensitive, [47]). Both complexes are required for certain functions such as proper expression of mid-late sporulation specific genes (e.g. spore wall formation), yet distinctly required, and specialized, for other operations [48]. The two module variants can be seen as a remarkable example of subfunctionalization.

RSC is an ATP-dependent chromatin remodeling complex of S. cerevisiae, essential for mitotic growth [43], that plays a role in expression regulation by activating and repressing the transcription [21]. This single complex exemplifies almost all aforementioned fates awaiting duplicated proteins (see Figure 6 and Table 4).

**Table 4:** Different fates of WGD paralogs involved in RSC complex.

WGD gene pair	Duplication type	Essential	Comments
RSC1/RSC2	intra-complex	n/n, synthetic lethal	mutually exclusive, both indispensable during fermentation, different deletion phenotypes

RSC3/RSC30	intra-complex	y/n	heterodimers, equal proportions, RSC30 duplicated post-WGD

RSC6/SNF12	bi-complex	n/n, phenotypic supression*	subfunctionalization

*) Phenotypic suppression of RSC6 and SNF11, an another SWI/SNF component, is revealed by the E-MAP assay [47].

Overhangs do not, unlike their paralogs, participate in a protein complex. Direct interaction data confirm that overhangs do not seem to be associated with their paralog's protein complex. In our opinion features such as lower fraction of essential proteins or faster sequence evolution, make overhangs likely to be cases of neofunctionalization, initially working under relaxed evolutionary constraints. We hypothesize that overhangs released from the control of the ancestral protein complex, which are not purged from the genome (such as aforementioned SSD1/DIS3 pair), may form "seeds" for emerging complexes. This, accompanied with draft of additional subunits may to form novel complexes and ultimately become more embedded in the core cellular machinery.

## Methods

### WGD, SSD gene sets and the assessment of the age of duplications

WGD paralogs, genes that duplicated at the time of whole-genome duplication were taken from [4]. SSD paralogs (Small Scale Duplications) represent the most recent, non-WGD gene duplications. The SSD list consists of best bi-directional hits, *i.e*., gene pairs (A, B), such that their alignment score is higher than alignments of A against any other gene in the genome, and higher than alignment of B with any other gene in the genome (self-alignments excluding). 87% of WGD genes pass the criterion of best bi-directional similarity and were excluded from the SSD dataset.

WGD and SSD types of paralogs both stem from the most recent duplication of a given gene. To determine whether SSD duplications preceded or followed the WGD event, it is enough to assess the phylogenetic distribution of paralogs in multiple fungi species. Using orthology data from [44] we established that SSD paralogs were present in two copies before the ancestor of yeast underwent the WGD event. More specifically, the analysis of the fungal gene trees [8] shows that among SSD paralogs which participate in complexes, out of 84 pairs only a single gene pair duplicated after the WGD event (RSC30/YHR054C) and only for eight SSD paralog pairs gene trees imply duplication after the divergence with *S. pombe *(see additional file 1, Table S5 and Methods).

### Ribosomal paralogs

From the initial analysis of our dataset of paralogs, it is apparent that genes involved in translation followed a distinct evolutionary route. Indeed, it is known from the literature that ribosomal protein sequences are highly constrained and many of the ribosomal protein pairs show exceptionally high levels of identity, likely subject to periodic gene conversion [20]. Interestingly, almost all cytoplasmic ribosomal proteins (CRPs), in a stark contrast to mitochondrial RP, were retained in duplicate after the WGD event. What could be the *raison d'être *this massive duplication? The set of CRP paralogs has many distinguishing properties, such as (a) a very similar amino acid sequence to paralogs, (b) a high mRNA expression correlation between paralogs (Spearman's correlation coefficient 0.8, see Methods for details), (c) the whole functional class, with few exceptions, was duplicated at WGD. These features imply a low level of functional differentiation and possibly an mRNA dosage increase as an explanation for the retention of both duplicates in CRPs, although new provocative evidence suggests more functional divergence than expected [45]. Nevertheless, such "Whole Ribosome Duplication" may signify the role the WGD event played in the evolution of anaerobic fermentation in yeast (compare with mitochondrial RP, additional file 1, Table S4).

Expression data were taken from the Gene Expression Omnibus (GEO) database [46] of the National Center for Biotechnology Information (NCBI), downloaded on 21 December 2006. Only multi-array datasets were considered, resulting in 357 microarray samples from 12 experiments, subsequently normalized (see additional file 1, Methods). We used Spearman's rank correlation coefficients to calculate the degree of co-expression between all gene pairs.

*K. waltii *orthologs were downloaded from Yeast Genome Order Browser (http://wolfe.gen.tcd.ie/browser[4]). Protein identity levels were calculated from alignments available in http://compbio.mit.edu.

Multiple module definitions were used to avoid bias of a certain protein complex annotation and make sure that results obtained hold independent of various protein complex maps used. MIPS data on protein complexes were downloaded from The MIPS Comprehensive Yeast Genome Database (CYGD, http://mips.gsf.de/[14]). The SGD GO complexes (in total 233 complexes, 1705 proteins) were generated by using the SGD GO component annotations (as of 9 May 2007) and then keeping only those components that have a GO description containing one of the following strings: complex, subunit, ribosome, proteasome, nucleosome, repairosome, degradosome, apoptosome, replisome, holoenzyme, snRNP. Only the lowest possible annotation level was maintained. Associations that where obtained from large scale experiments were removed.

## Authors' contributions

RS and BS designed the study. RS performed the analysis. MH contributed analytical methods. RS and BS wrote the manuscript. All authors read and approved the final manuscript.

## Supplementary Material

Additional file 1 Supplementary information. Supplementary information and additional tables. Click here for file
