# Supplementary material for: Complex fate of paralogs
Source: BMC Evol Biol. 2008 Dec 18;8:337. doi: 10.1186/1471-2148-8-337 (PMC2628386; doi:10.1186/1471-2148-8-337)
Supplement: Additional file 1 — Supplementary information. Supplementary information and additional tables. [file 1471-2148-8-337-S1.doc]

Supplementary Information

|  | SGD | MIPS | C&K | PICO |
| --- | --- | --- | --- | --- |
| WGD pairs | 27 | 27 | 49 | 55 |
| SSD pairs | 26 | 30 | 40 | 44 |
| Different* | No (P=0.97) | No (P=0.76) | No (P=0.35) | No (P=0.32) |

**Table SI1.** Intra-complex paralogs were analyzed for the two classes of duplication. For various protein complex maps numbers of paralog pairs where both proteins are part of the same module are shown. Cytosolic ribosomal proteins, all WGD paralogs, are excluded from the table. *) Different contributions to intra-complex class for the two types of duplication (WGD/SSD)

|  | SGD | MIPS | C&K | PICO |
| --- | --- | --- | --- | --- |
| WGD pairs | 5 | 2 | 26 | 6 |
| SSD pairs | 26 | 22 | 59 | 41 |
| Different* | Yes (P=0.0004) | Yes (P=0.0003) | Yes (P=0.0003) | Yes (P<2E6) |

**Table SI2.** *Different contributions to bi-complex class for the two types of duplication (WGD/SSD)* Various definitions of protein complex do not influence the directionality of the trend with SSD paralogs contributing significantly more to this class.

|  |  | # overhangs |  |  |
| --- | --- | --- | --- | --- |
|  | SGD | MIPS | C&K | PICO |
| WGD genes | 26 | 37 | 127 | 87 |
| SSD genes | 32 | 45 | 94 | 72 |
| Paralogs play a different role in modules* | No (P=0.41) | No (P=0.59) | Yes (P=0.01) | No (P=0.28) |

**Table SI3.** Manually-curated SGD protein complexes build based on GO categories.

|  | Cytoplasmic ribosomal proteins | Mitochondrial ribosomal proteins |
| --- | --- | --- |
| WGD paralogs | 110 | 0 |
| SSD paralogs | 8 | 0 |

**Table SI4** Contribution of two types of paralogs to the set of ribosomal proteins. Ribosomal protein counts after MIPS annotation. Two of the four SSD paralog pairs undergo gene conversion with amino acid identities of 98%.

| Gene 1 | | Gene 2 | | Duplication following the divergence with |
| --- | --- | --- | --- | --- |
| Name | Protein complex | Name | Protein complex |
| UBR1 | proteasome complex (sensu Eukaryota) | UBR2 |  | Y. lipolytica |
| NOT5 | CCR4-NOT core complex | NOT3 | CCR4-NOT core complex | C. albicans |
| AI5_ALPHA |  | COX1 | mitochondrial respiratory chain complex IV | S. bayanus |
| VAN1 | alpha-1,6-mannosyltransferase complex | ANP1 | alpha-1,6-mannosyltransferase complex | N. crassa |
| RSC30 | RSC complex | YHR054C |  | S. paradoxus |
| HTB2 | nuclear nucleosome | HTB1 | nuclear nucleosome | C. albicans |
| NGL1 |  | CCR4 | CCR4-NOT core complex, Cdc73/Paf1 complex | Y. lipolytica |
| REI1 | colocalizes with cytosolic large ribosomal subunit | REH1 |  | N. crassa |

**Table SI5** Timing of the duplication of the protein-complex associated SSD paralogs. Data from Wapinski et al., 2007


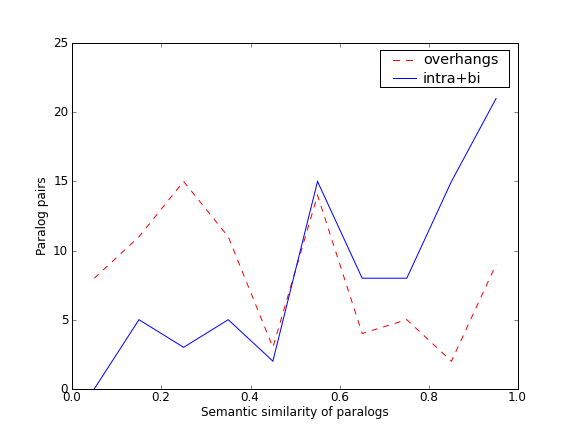


**Figure SI1.** Rapid functional post-duplication divergence of overhangs. X-axis denotes a semantic similarity of paralogs based on Gene Ontologies [9], and Y axis represents a number of paralog pairs. Compared to paralogs involved in complexes (solid blue line), a significant fraction of overhangs (dashed red line) show lower functional similarities.

### The RSC complex

Zinc-cluster domain containing RSC3 and RSC30 are paralogs found in RSC complexes in equal proportions (1) and are known to interact physically, forming a stable heteromeric complex (2). Despite operating as a heterodimer (possibly within the RSC complex as well), the two genes have different functions: RSC3 being an essential gene, while the RSC30 deficient mutant is viable yet osmosensitive (largely rescuable with an extra copy of RSC3). Nevertheless, the relationship between the two paralogs is, to a large extend, symmetric and even-though an additional copy of RSC30 does not rescue RSC3-deficient strains, multiple strains bearing serious RSC3 mutations can be alleviated by providing additional copies of RSC30 (2). Please note, that RSC30 duplicated yet again giving rise to a *S. cerevisiae* specific YHR054C gene.

### Tracing evolutionary past of paralogs

Among WGD duplicates only 5 pairs were found to be incorporated in different SGD complexes: RSC6/SNF12, DPB3/DLS1 (described in the text) and three other paralogous pairs involved in DNA metabolism. Sir2 (NAD+ dependent histone deacetylase of the Sirtuin family Imai et al. 2001) is involved in the regulation of lifespan and plays a role in silencing of a.o., telomeres (as nuclear telomere cap complex subunit) and RENT complex (rDNA silencing). Its WGD paralog, HST1, has conserved the property NAD+ histone deacetylation, and is an essential subunit of the Sum1p/Rfm1p/Hst1p complex required for ORC-dependent silencing (SGD). The fact that the overexpression of HST1 restores transcriptional silencing in a sir2 mutant (Brachmann et al. 1995, Xie et al. 1999), suggests strongly that the pre-WGD, ancient SIR2/HST1 protein was a versatile silencing protein. After the WGD event, HST1 assumed one of the roles of the ancestral protein [allowing SIR2 to focus on its complexes], while maintaining the ability to rescue SIR2-deficient strains.

### DNA Pol/CHRAC

Epsilon DNA polymerase is an essential complex that catalyzes processive DNA synthesis in the absence of PCNA, while the chromatin accessibility complex CHRAC is able to convert irregular chromatin into a regular array of nucleosomes (Figure fig-neosub-eps b). Unlike other known chromatin remodeling complexes, CHRAC can also function during chromatin assembly. The two complexes not only share a common subunit (DPB4, DNA polymerase II epsilon 4th subunit, Iida et al., 2003) but also contain a pair of WGD genes (see Figure fig-epsDNApol b). Involvement of DPB4 in both protein complexes strongly suggests that an ancestral protein DPB3/DLS1 was also common subunit of the two complexes in the pre-WGD yeast (Figure fig-epsDNApol a). The two proteins diverged significantly in their interaction partners following the duplication, and analysis of mass spectrometry data reveals a lack of interaction with the paralog's complex, i.e., with the exception of the shared subunit DPB4, DLS1 does not bind with the epsilon DNA polymerase subunits and DPB3 was never purified with components of CHRAC. Loss of interaction partners explains why deletion of both paralogs do not result in a synthetic growth defect (or even synthetic lethality), but what is surprising is that the DPB3/DLS1 genes undergo synthetic rescue, i.e. the deletion of both genes alleviates the genetic defect introduced by the deletion of only DLS1 gene (Iida et al., 2003).

## Supplementary Methods

Ohnologs, genes that duplicated at the time of whole-genome duplication and are preset in two copies in the yeast genome were taken from (3). This list is derived based on homology and conserved synteny methods. After preliminary analysis of ribosomes and their distinct mode of evolution (see Ribosomal Paralogs below), we decided to exclude paralogs involved in translation (mostly WGD paralogs) to uncover other, more subtle and non-biosynthetic signals in the dataset. This lead to a final list of 468 WGD paralogous pairs.

From the list of SSD paralogs we excluded very recent duplications, (more than 98% of amino acid identity), whose functional categories reveal identity of these genes as transposable elements (enriched for reverse transcriptase RT, protease PR and integrase IN), and excluded pairs which had no experimental evidence demonstrating the presence of gene product. We found almost all ohnologs (87%) on the SSD list and subsequently removed them to create distinct, non-overlapping dataset of 399 paralogs. We cannot exclude that some of the WGD genes are present on SSD list, due to e.g., genomic rearrangements obfuscating synteny recognition necessary to identify conserved synteny blocks.

Such reciprocal-best-hit method gives us an indication of the most recent duplication event; this approach was favored over all-against-all homology relationship, as it would bias our results toward large protein families (i.e., in a gene family with ten genes there are 45 homology relationships, while there were only nine duplications). Moreover, ancestral complex membership is even more difficult to deduce for older duplications, than for the recent ones

A bi-directional best hit approach allows us to focus on the most recent duplication per gene, avoiding a bias introduced if all pairwise homology relationships in a gene family were taken into account.

We assessed the impact of different types of duplication (WGD or SSD) on the fate of paralogs originating in complexes. We identified a differential involvement of paralogs in complexes; with only a minority of bi-complex paralogs stemming from WGD. We attribute the higher representation of SSD paralogs to the time of the duplications, as the majority of them preceded the WGD event. To determine the age of SSD paralogs with respect to WGD event, we made use of homology data for multiple fungi species (4). We find that 80% of gene pairs with identified gene products have different homologs in one of the species which diverged before the WGD event: *A. gossypii, K. lactis, K. waltii, S. kluyveri*. As both gene copies were present in the genome of the ancestor of *S. cereviseae* at the time of whole-genome duplication, we can conclude that even SSD duplications can be thought of as the most recent, a great majority of them is certainly not young.

### Null model of complex/paralog membership

To estimate the statistical significance of numbers of paralogs in different paralog classes (intra-complex, bi-complex) we designed a simple null model scenario of protein complex membership. With paralogy relationships between protein pairs being preserved, modules are reshuffled. A 1-1 mapping between a protein and a randomly chosen other protein from the same set is created. A new set of modules is created, with old proteins replaced by their counterparts according to the mapping. This preserves the module structure (number and size of modules) and does not change the number of proteins participating in multiple modules. The reshuffling is repeated 10000 times to derive the average number of bi-complex and intra-complex paralogs.

### Spoke value calculation

To measure direct protein-protein interactions we used the spoke mathematical model (5), which measures the tendency for proteins to identify each other when tagged.


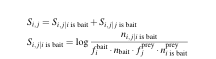


where *S_i,j_*_|_*_i_* _is bait_ is the number of times the protein i was retrieved when i is tagged. n_bait_ is a total number of purifications. f^bait^_i_ is the fraction of purification where protein i was bait. f^prey^_j_ is the fraction of all retrieved preys that were protein j. n^prey^_i is bait_ is the number of preys retrieved with protein i as bait.

The data for the model consisted of merged datasets of (5, 6). Based on this data we calculated direct interaction scores between all protein pairs. Intra complex paralogs with a spoke value greater or equal to 5.0 were classified as paralog addition (both proteins physically present within the same protein complex). Paralogs with spoke less or equal to 0.0 (or never purified together) were considered module variants.

### Overhangs

The analysis of functional divergence of overhangs was based on MIPS data, not SGD complexes as most of the other analyses. This step was necessary to avoid the circular reasoning possibly introduced by deriving complexes from GO SGD data and, at the same time, testing their functional divergence using GO. The semantic similarity measure was implemented after [9]. Figure SI1 shows rapid divergence of functionality for overhangs (median semantic similarity of 0.34) compared to the two other type of paralogs, intra- and bi-complex (median 0.73, one-sided Wilcoxon test P<1e-7). This analysis was repeated using mass-spectrometry-derived complexes (one-sided Wilcoxon test, P<2e-4 for PICO and P<2e-2 for C&K complexes).

### Timing of the SSD gene gene duplication

The number of SSD paralog paris involved in protein complexes is 84. Analysis of gene trees of fungal orthogroups (Wapinski et al., 2007) shows that among these, only a single paralog pair is younger than the WGD event (RSC30/YHR054C and this case is described in the text). One may look for duplications only slightly older than the WGD event (following the yeast divergence with *C. albicans* lineage but before the WGD event). The analysis of fungal gene trees shows only two of 84 SSD paralog pairs involved in protein complexes duplicated within this time frame (HTB1/HTB2 and NOT3/NOT5). Considering duplications following the divergence with *S. pombe*, there are eight protein complex-associated paralog pairs in the SSD dataset (Table SI5). By identifying *S. pombe* orthologs of yeast genes (http://www.genedb.org/genedb/pombe/) we confirm that the large part of our SSD paralogs originated before the divergence with S. pombe (i.e., is older than ~1bln years compared to ~100mln years for the WGD event). We note a strong bias toward ancient duplications (76 vs. 8) in the SSD dataset.

### Protein complex data

PICO computationally derived complexes were downloaded from supplementary information of (7). C&K complexes build based on the pairwise protein score from (8) and were obtained from Patrick Kemmeren.

1. Cairns BR*, et al.* (1996) RSC, an essential, abundant chromatin-remodeling complex. *Cell* 87:1249-1260.

2. Angus-Hill ML*, et al.* (2001) A Rsc3/Rsc30 Zinc Cluster Dimer Reveals Novel Roles for the Chromatin Remodeler RSC in Gene Expression and Cell Cycle Control. *Molecular Cell* 7:741-751.

3. Byrne KP, Wolfe KH (2005) The Yeast Gene Order Browser: Combining curated homology and syntenic context reveals gene fate in polyploid species. *Genome Res* 15:1456-1461.

4. Scannell DR, Byrne KP, Gordon JL, Wong S, Wolfe KH (2006) Multiple rounds of speciation associated with reciprocal gene loss in polyploid yeasts. *Nature* 440:341-345.

5. Gavin A-C*, et al.* (2006) Proteome survey reveals modularity of the yeast cell machinery. *Nature* 440:631-636.

6. Krogan NJ*, et al.* (2006) Global landscape of protein complexes in the yeast Saccharomyces cerevisiae. *Nature* 440:637-643.

7. Hart GT, Lee I, Marcotte E (2007) A high-accuracy consensus map of yeast protein complexes reveals modular nature of gene essentiality. *BMC Bioinformatics* 8:236.

1. Collins SR*, et al.* (2007) Toward a comprehensive atlas of the physical interactome of Saccharomyces cerevisiae. *Molecular & cellular proteomics: MCP* 6:439-450.
2. Tao Y et al. (2007) Information theory applied to the sparse gene ontology annotation network to predict novel gene function. Bioinformatics 23:i529-538.

### Expression data

Expression data were taken from the Gene Expression Omnibus (GEO) database (Edgar, Domrachev et al. 2002) of the National Center for Biotechnology Information (NCBI), downloaded on 21 december 2006. In order to maximize consistency of the expression data, only data based on the Affymetrix GeneChip Yeast Genome S98 Array YG-S98 expression platform (GEO platform GPL90) were used. Only experiments involving at least 10 microarray samples for which the raw signal intensity data were available were considered, resulting in 357 microarray samples from 12 experiments (Table SI5). Only the 6563 yeast genes with systematic names were considered. Where there were multiple probe sets per gene the median signal intensity value was taken.

Within-array normalization was done using the Robust Multi-array Averaging (RMA) algorithm (Irizarry, Hobbs et al. 2003) as implemented in the R statistical software bioconductor library (Gentleman, Carey et al. 2004). However, between the arrays expression values were normalized according to total array expression level. This was done by dividing the expression values by the mean array expression value. This prevents spurious correlation due to differences in total expression level between arrays, and yielded better results than between-array RMA normalization in other co-expression analyses (data not shown).

We used Spearman rank correlation coefficients to calculate the degree of co-expression between all gene pairs, as the microarray expression values were not normally distributed. Correlation coefficients were calculated per experiment and the mean value over the experiments was taken as the final co-expression score.

GEO series ID    Number of samples    Reference

GSE4807    30    (Tai, Boer et al. 2005)

GSE6073    12    (Yarragudi, Parfrey et al. 2007)

GSE1311, GSE1312, GSE1313    66    (Singh, Kumar et al. 2005)

GSE1639    18    (Sabet, Volo et al. 2004)

GSE1693    26    (Hochwagen, Wrobel et al. 2005)

GSE1934    24    (Schawalder, Kabani et al. 2004)

GSE1938    15    (Pitkanen, Torma et al. 2004)

GSE1975    28    (Ronald, Akey et al. 2005)

GSE2343    12    (Takagi, Masuda et al. 2005)

GSE3076    96    (Guan, Zheng et al. 2006)

GSE3821    16    (Kresnowati, van Winden et al. 2006)

GSE4135    14    (Yu, Palumbo et al. 2006)

**Table SI5.** Expression data sets used.

References

Edgar, R., M. Domrachev, et al. (2002). "Gene Expression Omnibus: NCBI gene expression and hybridization array data repository." Nucleic Acids Res 30(1): 207-10.

Gentleman, R. C., V. J. Carey, et al. (2004). "Bioconductor: open software development for computational biology and bioinformatics." Genome Biol 5(10): R80.

Guan, Q., W. Zheng, et al. (2006). "Impact of nonsense-mediated mRNA decay on the global expression profile of budding yeast." PLoS Genet 2(11): e203.

Hochwagen, A., G. Wrobel, et al. (2005). "Novel response to microtubule perturbation in meiosis." Mol Cell Biol 25(11): 4767-81.

Irizarry, R. A., B. Hobbs, et al. (2003). "Exploration, normalization, and summaries of high density oligonucleotide array probe level data." Biostatistics 4(2): 249-64.

Kresnowati, M. T., W. A. van Winden, et al. (2006). "When transcriptome meets metabolome: fast cellular responses of yeast to sudden relief of glucose limitation." Mol Syst Biol 2: 49.

Pitkanen, J. P., A. Torma, et al. (2004). "Excess mannose limits the growth of phosphomannose isomerase PMI40 deletion strain of Saccharomyces cerevisiae." J Biol Chem 279(53): 55737-43.

Ronald, J., J. M. Akey, et al. (2005). "Simultaneous genotyping, gene-expression measurement, and detection of allele-specific expression with oligonucleotide arrays." Genome Res 15(2): 284-91.

Sabet, N., S. Volo, et al. (2004). "Genome-wide analysis of the relationship between transcriptional regulation by Rpd3p and the histone H3 and H4 amino termini in budding yeast." Mol Cell Biol 24(20): 8823-33.

Schawalder, S. B., M. Kabani, et al. (2004). "Growth-regulated recruitment of the essential yeast ribosomal protein gene activator Ifh1." Nature 432(7020): 1058-61.

Singh, J., D. Kumar, et al. (2005). "Transcriptional response of Saccharomyces cerevisiae to desiccation and rehydration." Appl Environ Microbiol 71(12): 8752-63.

Tai, S. L., V. M. Boer, et al. (2005). "Two-dimensional transcriptome analysis in chemostat cultures. Combinatorial effects of oxygen availability and macronutrient limitation in Saccharomyces cerevisiae." J Biol Chem 280(1): 437-47.

Takagi, Y., C. A. Masuda, et al. (2005). "Ubiquitin ligase activity of TFIIH and the transcriptional response to DNA damage." Mol Cell 18(2): 237-43.

Yarragudi, A., L. W. Parfrey, et al. (2007). "Genome-wide analysis of transcriptional dependence and probable target sites for Abf1 and Rap1 in Saccharomyces cerevisiae." Nucleic Acids Res 35(1): 193-202.

Yu, C., M. J. Palumbo, et al. (2006). "Contribution of the histone H3 and H4 amino termini to Gcn4p- and Gcn5p-mediated transcription in yeast." J Biol Chem 281(14): 9755-64.
